# 2-Meth­oxy­ethanaminium periodate 18-crown-6 clathrate

**DOI:** 10.1107/S1600536811002315

**Published:** 2011-01-22

**Authors:** Jin-Gang Hu

**Affiliations:** a Ordered Matter Science Research Center, Southeast University, Nanjing 210096, People’s Republic of China

## Abstract

In the crystal structure of the title organic salt, C_3_H_10_NO^+^·IO_4_^−^·C_12_H_24_O_6_, the protonated 2-meth­oxy­ethanaminium (CH_3_OC_2_H_4_—NH_3_^+^) cation forms a 1:1 supra­molecular rotator–stator complex with the 18-crown-6 mol­ecule *via* N—H⋯O hydrogen bonds. The (CH_3_OC_2_H_4_—NH_3_^+^) group is attached from the convex side of the bowl-shaped crown, in contrast to similar ammonium cations that nest in the curvature of the bowl. The cations are associated *via* N—H⋯O inter­actions, while the cations and anions are linked by weak C—H⋯O hydrogen bonds, forming cation–crown–anion chains parallel to [010].

## Related literature

For the use of crown ethers in catalysis, solvent extraction, isotope separation, bionics, materials chemistry, host–guest chemistry and supra­molecular chemistry, see: Clark *et al.* (1998 ▶); Nakamura *et al.* (1998 ▶). For their ability to form non-covalent hydrogen-bonded complexes with ammonium cations, both in the solid state and in solution, see: Fender *et al.* (2002 ▶); Kryatova *et al.* (2004 ▶). Various types of *R*NH_3_^+^ structures (*R* = H, CH_3_, C_6_H_5_CH_2_, NH_2_, *etc*.) have been shown to form stable ammonium crown ether complexes in the solid state, see: Akutagawa *et al.* (2005 ▶, 2009 ▶). For a related structure, see: Fu *et al.* (2010 ▶). 
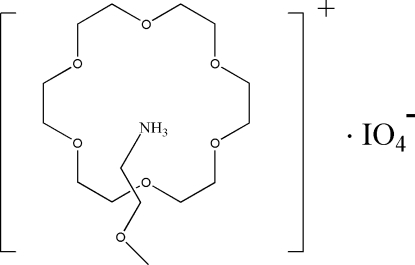


## Experimental

### 

#### Crystal data

C_3_H_10_NO^+^·IO_4_^−^·C_12_H_24_O_6_
*M* *_r_* = 531.33
Monoclinic, 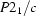

*a* = 13.118 (3) Å
*b* = 8.4229 (17) Å
*c* = 22.176 (7) Å
β = 111.81 (3)°
*V* = 2274.9 (10) Å^3^
*Z* = 4
Mo *K*α radiation
μ = 1.46 mm^−1^
*T* = 293 K
0.20 × 0.20 × 0.20 mm

#### Data collection

Rigaku SCXmini diffractometer
Absorption correction: multi-scan (*CrystalClear*; Rigaku/MSC, 2005 ▶) *T*_min_ = 0.747, *T*_max_ = 0.754
22779 measured reflections
5201 independent reflections
4138 reflections with *I* > 2σ(*I*)
*R*_int_ = 0.067

#### Refinement

*R*[*F* ^2^ > 2σ(*F* ^2^)] = 0.054
*wR*(*F* ^2^) = 0.146
*S* = 1.05
5201 reflections
253 parameters
H-atom parameters constrained
Δρ_max_ = 1.74 e Å^−3^
Δρ_min_ = −1.26 e Å^−3^

### 

Data collection: *CrystalClear* (Rigaku/MSC, 2005 ▶); cell refinement: *CrystalClear*; data reduction: *CrystalClear*; program(s) used to solve structure: *SHELXS97* (Sheldrick, 2008 ▶); program(s) used to refine structure: *SHELXL97* (Sheldrick, 2008 ▶); molecular graphics: *SHELXTL* (Sheldrick, 2008 ▶); software used to prepare material for publication: *SHELXL97*.

## Supplementary Material

Crystal structure: contains datablocks I, global. DOI: 10.1107/S1600536811002315/vm2071sup1.cif

Structure factors: contains datablocks I. DOI: 10.1107/S1600536811002315/vm2071Isup2.hkl

Additional supplementary materials:  crystallographic information; 3D view; checkCIF report

## Figures and Tables

**Table 1:** Hydrogen-bond geometry (Å, °)

*D*—H⋯*A*	*D*—H	H⋯*A*	*D*⋯*A*	*D*—H⋯*A*
N1—H1*C*⋯O1	0.89	2.30	3.000 (4)	135
N1—H1*C*⋯O2	0.89	2.15	2.912 (4)	144
N1—H1*D*⋯O3	0.89	2.43	2.980 (5)	121
N1—H1*D*⋯O4	0.89	2.03	2.886 (4)	161
N1—H1*E*⋯O5	0.89	2.43	3.010 (5)	123
N1—H1*E*⋯O6	0.89	2.06	2.870 (4)	151
C1—H1*B*⋯O11^i^	0.97	2.54	3.398 (9)	148
C13—H13*A*⋯O8	0.97	2.52	3.288 (7)	135
C13—H13*B*⋯O1^ii^	0.97	2.54	3.504 (6)	171
C15—H15*B*⋯O10^iii^	0.96	2.47	3.370 (9)	156

Symmetry codes: (i) 
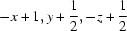
; (ii) 
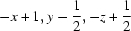
; (iii) 
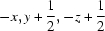
.

**Fig. 1.**
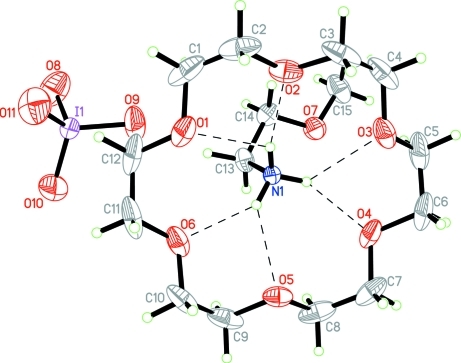
The molecular structure of the title compound, with the atomic numbering scheme (H atom labels have been omitted for clarity). Displacement ellipsoids are drawn at the 30% probability level, and N—H···O hydrogen bonds are shown as dotted lines.

**Fig. 2.**
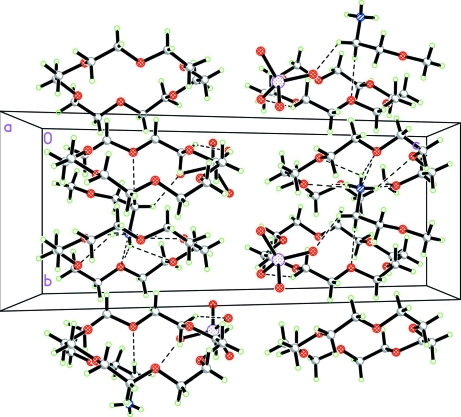
Crystal packing of the title compound viewed along the a axis showing the N—H···O and, C—H···O interactions (dotted lines).

## supplementary crystallographic information

### Comment

The ability of 18-crown-6 ether (18-C-6) to form complexes with different metal ions and organic proton donors has been widely investigated. Because of their novel coordination modes, crown ethers have been widely used in catalysis, solvent extraction, isotope separation, bionics, materials chemistry, host-guest chemistry and supramolecular chemistry (Clark *et al.*, 1998; Nakamura *et al.*, 1998). Crown ethers recnetly have attracted much attention due to their ability to form non-covalent hydrogen-bonded complexes with ammonium cations, both in the solid state and in solution (Fender *et al.,* 2002; Kryatova *et al.*, 2004). The structures of organic ammonium RNH_3_^+^.crown ether assemblies in the solid state depend not only on the structure of the cation and the size of the crown ether ring, but also on the nature of the counter-anion. Various types of RNH_3_^+ ^structures (R =H, CH_3_, C_6_H_5_CH_2_, NH_2_, etc.) have been shown to form stable ammonium.crown ether complexes in the solid state (Akutagawa *et al.,* 2005, 2009).

We report here the crystal structure of 2-methoxy-ethylamine periodate 18-crown-6 clathrate. X-ray crystallographic studies have been carried out for the complex C_3_H_9_NO^+.^IO_4_^-.^C_12_H_24_O_6_ at room temperature. An view of the complex is shown in Fig. 1. The ionic radius of NH_3_^+^ matches the cavity size of six-O crown ethers, and N—H···O hydrogen bonds (Table 1) help to form stable NH_3_^+^.crown ether complexes, displayed in Fig. 2.

### Experimental

2-Methoxy-ethylamine (1.50 g, 0.02 mol), 18-crown-6 (5.28 g, 0.02 mol) and HIO_4_ (4.56 g, 0.02 mol) were dissolved in 30 ml ethanol. Colorless s//ingle crystals of the title compound suitable for X-ray analysis were obtained via slow evaporation of the solvent at room temperature over a period of 3 days.

### Refinement

Hydrogen atom positions were calculated and allowed to ride on their respective C atoms and N atoms with C–H distances of 0.93–0.97Å and N–H = 0.86 Å, and with U_iso_(H)=1.2U_eq_(C or N).

### Crystal data

**Table:**

C_3_H_10_NO^+^·IO_4_^−^·C_12_H_24_O_6_	*F*(000) = 1088
*M**_r_* = 531.33	*D*_x_ = 1.551 Mg m^−^^3^
Monoclinic, *P*2_1_/*c*	Mo *K*α radiation, λ = 0.71073 Å
Hall symbol: -P 2ybc	Cell parameters from 121476 reflections
*a* = 13.118 (3) Å	θ = 3.0–27.5°
*b* = 8.4229 (17) Å	µ = 1.46 mm^−^^1^
*c* = 22.176 (7) Å	*T* = 293 K
β = 111.81 (3)°	Prism, white
*V* = 2274.9 (10) Å^3^	0.20 × 0.20 × 0.20 mm
*Z* = 4	

### Data collection

**Table:**

Rigaku SCXmini diffractometer	5201 independent reflections
Radiation source: fine-focus sealed tube	4138 reflections with *I* > 2σ(*I*)
graphite	*R*_int_ = 0.067
Detector resolution: 13.6612 pixels mm^-1^	θ_max_ = 27.5°, θ_min_ = 3.0°
CCD_Profile_fitting scans	*h* = −17→17
Absorption correction: multi-scan (*CrystalClear*; Rigaku/MSC, 2005)	*k* = −10→10
*T*_min_ = 0.747, *T*_max_ = 0.754	*l* = −28→28
22779 measured reflections	

### Refinement

**Table:**

Refinement on *F*^2^	Primary atom site location: structure-invariant direct methods
Least-squares matrix: full	Secondary atom site location: difference Fourier map
*R*[*F*^2^ > 2σ(*F*^2^)] = 0.054	Hydrogen site location: inferred from neighbouring sites
*wR*(*F*^2^) = 0.146	H-atom parameters constrained
*S* = 1.05	*w* = 1/[σ^2^(*F*_o_^2^) + (0.0576*P*)^2^ + 3.350*P*] where *P* = (*F*_o_^2^ + 2*F*_c_^2^)/3
5201 reflections	(Δ/σ)_max_ = 0.001
253 parameters	Δρ_max_ = 1.74 e Å^−^^3^
0 restraints	Δρ_min_ = −1.26 e Å^−^^3^

### Special details

**Table:**

Geometry. All esds (except the esd in the dihedral angle between two l.s. planes) are estimated using the full covariance matrix. The cell esds are taken into account individually in the estimation of esds in distances, angles and torsion angles; correlations between esds in cell parameters are only used when they are defined by crystal symmetry. An approximate (isotropic) treatment of cell esds is used for estimating esds involving l.s. planes.
Refinement. Refinement of F^2^ against ALL reflections. The weighted R-factor wR and goodness of fit S are based on F^2^, conventional R-factors R are based on F, with F set to zero for negative F^2^. The threshold expression of F^2^ > 2sigma(F^2^) is used only for calculating R-factors(gt) etc. and is not relevant to the choice of reflections for refinement. R-factors based on F^2^ are statistically about twice as large as those based on F, and R- factors based on ALL data will be even larger.

### Fractional atomic coordinates and isotropic or equivalent isotropic displacement parameters (Å^2^)

**Table:**

	*x*	*y*	*z*	*U*_iso_*/*U*_eq_	
C1	0.5224 (5)	0.6622 (8)	0.1488 (3)	0.0852 (19)	
H1A	0.5451	0.7725	0.1528	0.102*	
H1B	0.5460	0.6124	0.1167	0.102*	
C2	0.5742 (5)	0.5819 (7)	0.2115 (4)	0.088 (2)	
H2A	0.5480	0.4733	0.2081	0.106*	
H2B	0.6531	0.5792	0.2231	0.106*	
C3	0.6022 (4)	0.5926 (7)	0.3233 (3)	0.088 (2)	
H3A	0.6812	0.5962	0.3353	0.106*	
H3B	0.5805	0.4822	0.3222	0.106*	
C4	0.5719 (5)	0.6786 (7)	0.3718 (3)	0.089 (2)	
H4A	0.6127	0.6368	0.4148	0.107*	
H4B	0.5898	0.7903	0.3714	0.107*	
C5	0.4229 (8)	0.7282 (8)	0.4040 (3)	0.101 (3)	
H5A	0.4449	0.8388	0.4105	0.121*	
H5B	0.4571	0.6731	0.4450	0.121*	
C6	0.3006 (8)	0.7164 (8)	0.3824 (3)	0.097 (2)	
H6A	0.2781	0.6061	0.3748	0.117*	
H6B	0.2770	0.7578	0.4159	0.117*	
C7	0.1360 (6)	0.8074 (10)	0.3024 (4)	0.102 (3)	
H7A	0.1129	0.8481	0.3362	0.122*	
H7B	0.1072	0.7007	0.2916	0.122*	
C8	0.0934 (5)	0.9077 (9)	0.2455 (4)	0.100 (2)	
H8A	0.0147	0.9201	0.2330	0.120*	
H8B	0.1270	1.0119	0.2552	0.120*	
C9	0.0830 (4)	0.9315 (7)	0.1374 (3)	0.092 (2)	
H9A	0.1256	1.0288	0.1459	0.110*	
H9B	0.0063	0.9596	0.1252	0.110*	
C10	0.0982 (5)	0.8449 (8)	0.0838 (3)	0.091 (2)	
H10A	0.0604	0.7437	0.0774	0.109*	
H10B	0.0672	0.9057	0.0439	0.109*	
C11	0.2322 (7)	0.7290 (8)	0.0503 (3)	0.096 (2)	
H11A	0.1953	0.7769	0.0079	0.115*	
H11B	0.2039	0.6222	0.0492	0.115*	
C12	0.3523 (7)	0.7235 (8)	0.0657 (3)	0.093 (2)	
H12A	0.3674	0.6618	0.0330	0.111*	
H12B	0.3801	0.8302	0.0656	0.111*	
C13	0.2704 (4)	0.4729 (5)	0.2349 (2)	0.0524 (10)	
H13A	0.2642	0.4720	0.2772	0.063*	
H13B	0.3262	0.3962	0.2360	0.063*	
C14	0.1627 (4)	0.4234 (5)	0.1843 (2)	0.0554 (10)	
H14A	0.1361	0.3280	0.1982	0.066*	
H14B	0.1086	0.5067	0.1780	0.066*	
C15	0.0806 (5)	0.3509 (7)	0.0747 (3)	0.0828 (17)	
H15A	0.0963	0.3330	0.0362	0.124*	
H15B	0.0274	0.4344	0.0667	0.124*	
H15C	0.0518	0.2553	0.0859	0.124*	
I1	0.22495 (3)	0.22656 (4)	0.422019 (14)	0.06214 (15)	
N1	0.3057 (2)	0.6331 (4)	0.22280 (14)	0.0400 (7)	
H1C	0.3701	0.6565	0.2537	0.060*	
H1D	0.2559	0.7045	0.2232	0.060*	
H1E	0.3123	0.6344	0.1843	0.060*	
O1	0.5496 (2)	0.6619 (4)	0.26073 (18)	0.0613 (8)	
O2	0.4587 (3)	0.6605 (4)	0.35678 (15)	0.0729 (10)	
O3	0.2523 (3)	0.8040 (5)	0.32505 (18)	0.0730 (10)	
O4	0.1168 (3)	0.8372 (4)	0.1939 (2)	0.0724 (10)	
O5	0.2119 (3)	0.8196 (4)	0.09849 (15)	0.0719 (10)	
O6	0.4055 (3)	0.6534 (5)	0.12774 (16)	0.0705 (9)	
O7	0.1787 (3)	0.3952 (5)	0.12675 (15)	0.0679 (9)	
O8	0.2020 (5)	0.2732 (6)	0.3416 (2)	0.1104 (17)	
O9	0.1995 (5)	0.3899 (6)	0.4627 (3)	0.1233 (18)	
O10	0.1278 (5)	0.0779 (7)	0.4205 (3)	0.132 (2)	
O11	0.3531 (5)	0.1407 (11)	0.4607 (3)	0.161 (3)	

### Atomic displacement parameters (Å^2^)

**Table:**

	*U*^11^	*U*^22^	*U*^33^	*U*^12^	*U*^13^	*U*^23^
C1	0.096 (4)	0.079 (4)	0.117 (5)	−0.020 (3)	0.082 (4)	−0.032 (4)
C2	0.061 (3)	0.061 (3)	0.162 (7)	0.000 (3)	0.063 (4)	−0.021 (4)
C3	0.044 (3)	0.065 (3)	0.126 (5)	0.008 (2)	−0.003 (3)	0.024 (3)
C4	0.078 (4)	0.070 (3)	0.072 (3)	0.001 (3)	−0.026 (3)	0.012 (3)
C5	0.165 (8)	0.088 (4)	0.032 (2)	−0.017 (4)	0.016 (3)	−0.002 (2)
C6	0.165 (8)	0.096 (4)	0.057 (3)	−0.031 (4)	0.072 (4)	−0.017 (3)
C7	0.091 (5)	0.116 (5)	0.136 (7)	−0.032 (4)	0.085 (5)	−0.058 (5)
C8	0.060 (3)	0.097 (5)	0.149 (7)	0.001 (3)	0.046 (4)	−0.050 (5)
C9	0.056 (3)	0.056 (3)	0.121 (5)	0.012 (2)	−0.016 (3)	0.008 (3)
C10	0.080 (4)	0.074 (4)	0.070 (3)	−0.008 (3)	−0.028 (3)	0.016 (3)
C11	0.145 (7)	0.095 (4)	0.034 (3)	−0.042 (4)	0.018 (3)	−0.005 (2)
C12	0.153 (7)	0.093 (4)	0.048 (3)	−0.043 (4)	0.055 (4)	−0.012 (3)
C13	0.068 (3)	0.043 (2)	0.042 (2)	0.0030 (19)	0.0155 (19)	0.0039 (17)
C14	0.063 (3)	0.045 (2)	0.064 (3)	−0.0082 (19)	0.030 (2)	−0.0042 (19)
C15	0.092 (4)	0.068 (3)	0.063 (3)	−0.020 (3)	−0.002 (3)	−0.002 (3)
I1	0.0582 (2)	0.0813 (3)	0.0510 (2)	0.00434 (14)	0.02498 (15)	0.00660 (14)
N1	0.0372 (15)	0.0504 (18)	0.0316 (14)	0.0007 (13)	0.0120 (12)	−0.0011 (12)
O1	0.0419 (15)	0.0467 (16)	0.091 (2)	0.0054 (13)	0.0197 (15)	−0.0005 (16)
O2	0.086 (2)	0.073 (2)	0.0412 (17)	−0.016 (2)	0.0025 (16)	−0.0003 (16)
O3	0.090 (3)	0.077 (2)	0.069 (2)	−0.0169 (19)	0.050 (2)	−0.0153 (18)
O4	0.0525 (18)	0.0593 (19)	0.098 (3)	0.0103 (16)	0.0192 (18)	−0.0143 (19)
O5	0.083 (2)	0.070 (2)	0.0427 (17)	−0.0211 (19)	−0.0002 (16)	0.0024 (15)
O6	0.089 (2)	0.081 (2)	0.0587 (19)	−0.028 (2)	0.0482 (18)	−0.0149 (17)
O7	0.0594 (19)	0.090 (2)	0.0497 (17)	−0.0077 (17)	0.0150 (14)	−0.0147 (16)
O8	0.148 (5)	0.121 (4)	0.061 (3)	−0.016 (3)	0.037 (3)	0.022 (2)
O9	0.157 (5)	0.095 (3)	0.121 (4)	0.005 (3)	0.056 (4)	−0.032 (3)
O10	0.161 (5)	0.111 (4)	0.159 (5)	−0.040 (4)	0.101 (4)	−0.006 (4)
O11	0.096 (4)	0.278 (9)	0.102 (4)	0.072 (5)	0.030 (3)	0.018 (5)

### Geometric parameters (Å, °)

**Table:**

C1—O6	1.429 (7)	C9—H9A	0.9700
C1—C2	1.468 (9)	C9—H9B	0.9700
C1—H1A	0.9700	C10—O5	1.420 (7)
C1—H1B	0.9700	C10—H10A	0.9700
C2—O1	1.418 (7)	C10—H10B	0.9700
C2—H2A	0.9700	C11—O5	1.417 (7)
C2—H2B	0.9700	C11—C12	1.484 (11)
C3—O1	1.426 (7)	C11—H11A	0.9700
C3—C4	1.468 (9)	C11—H11B	0.9700
C3—H3A	0.9700	C12—O6	1.420 (7)
C3—H3B	0.9700	C12—H12A	0.9700
C4—O2	1.405 (7)	C12—H12B	0.9700
C4—H4A	0.9700	C13—N1	1.484 (5)
C4—H4B	0.9700	C13—C14	1.500 (6)
C5—O2	1.416 (8)	C13—H13A	0.9700
C5—C6	1.497 (11)	C13—H13B	0.9700
C5—H5A	0.9700	C14—O7	1.390 (5)
C5—H5B	0.9700	C14—H14A	0.9700
C6—O3	1.401 (8)	C14—H14B	0.9700
C6—H6A	0.9700	C15—O7	1.424 (6)
C6—H6B	0.9700	C15—H15A	0.9600
C7—O3	1.418 (8)	C15—H15B	0.9600
C7—C8	1.446 (11)	C15—H15C	0.9600
C7—H7A	0.9700	I1—O11	1.736 (5)
C7—H7B	0.9700	I1—O8	1.740 (4)
C8—O4	1.421 (7)	I1—O9	1.744 (5)
C8—H8A	0.9700	I1—O10	1.778 (5)
C8—H8B	0.9700	N1—H1C	0.8900
C9—O4	1.409 (7)	N1—H1D	0.8900
C9—C10	1.470 (9)	N1—H1E	0.8900
			
O6—C1—C2	110.4 (4)	O5—C10—H10A	109.8
O6—C1—H1A	109.6	C9—C10—H10A	109.8
C2—C1—H1A	109.6	O5—C10—H10B	109.8
O6—C1—H1B	109.6	C9—C10—H10B	109.8
C2—C1—H1B	109.6	H10A—C10—H10B	108.2
H1A—C1—H1B	108.1	O5—C11—C12	108.9 (5)
O1—C2—C1	110.7 (4)	O5—C11—H11A	109.9
O1—C2—H2A	109.5	C12—C11—H11A	109.9
C1—C2—H2A	109.5	O5—C11—H11B	109.9
O1—C2—H2B	109.5	C12—C11—H11B	109.9
C1—C2—H2B	109.5	H11A—C11—H11B	108.3
H2A—C2—H2B	108.1	O6—C12—C11	109.5 (5)
O1—C3—C4	110.2 (4)	O6—C12—H12A	109.8
O1—C3—H3A	109.6	C11—C12—H12A	109.8
C4—C3—H3A	109.6	O6—C12—H12B	109.8
O1—C3—H3B	109.6	C11—C12—H12B	109.8
C4—C3—H3B	109.6	H12A—C12—H12B	108.2
H3A—C3—H3B	108.1	N1—C13—C14	112.8 (3)
O2—C4—C3	108.9 (4)	N1—C13—H13A	109.0
O2—C4—H4A	109.9	C14—C13—H13A	109.0
C3—C4—H4A	109.9	N1—C13—H13B	109.0
O2—C4—H4B	109.9	C14—C13—H13B	109.0
C3—C4—H4B	109.9	H13A—C13—H13B	107.8
H4A—C4—H4B	108.3	O7—C14—C13	108.2 (4)
O2—C5—C6	110.3 (5)	O7—C14—H14A	110.1
O2—C5—H5A	109.6	C13—C14—H14A	110.1
C6—C5—H5A	109.6	O7—C14—H14B	110.1
O2—C5—H5B	109.6	C13—C14—H14B	110.1
C6—C5—H5B	109.6	H14A—C14—H14B	108.4
H5A—C5—H5B	108.1	O7—C15—H15A	109.5
O3—C6—C5	108.9 (5)	O7—C15—H15B	109.5
O3—C6—H6A	109.9	H15A—C15—H15B	109.5
C5—C6—H6A	109.9	O7—C15—H15C	109.5
O3—C6—H6B	109.9	H15A—C15—H15C	109.5
C5—C6—H6B	109.9	H15B—C15—H15C	109.5
H6A—C6—H6B	108.3	O11—I1—O8	111.6 (3)
O3—C7—C8	109.7 (5)	O11—I1—O9	114.1 (3)
O3—C7—H7A	109.7	O8—I1—O9	111.0 (3)
C8—C7—H7A	109.7	O11—I1—O10	105.8 (4)
O3—C7—H7B	109.7	O8—I1—O10	106.8 (3)
C8—C7—H7B	109.7	O9—I1—O10	107.0 (3)
H7A—C7—H7B	108.2	C13—N1—H1C	109.5
O4—C8—C7	109.2 (5)	C13—N1—H1D	109.5
O4—C8—H8A	109.8	H1C—N1—H1D	109.5
C7—C8—H8A	109.8	C13—N1—H1E	109.5
O4—C8—H8B	109.8	H1C—N1—H1E	109.5
C7—C8—H8B	109.8	H1D—N1—H1E	109.5
H8A—C8—H8B	108.3	C2—O1—C3	112.8 (5)
O4—C9—C10	110.3 (4)	C4—O2—C5	113.1 (5)
O4—C9—H9A	109.6	C6—O3—C7	113.4 (6)
C10—C9—H9A	109.6	C9—O4—C8	113.0 (5)
O4—C9—H9B	109.6	C10—O5—C11	112.5 (5)
C10—C9—H9B	109.6	C12—O6—C1	112.1 (5)
H9A—C9—H9B	108.1	C14—O7—C15	113.0 (4)
O5—C10—C9	109.6 (4)		

### Hydrogen-bond geometry (Å, °)

**Table:**

*D*—H···*A*	*D*—H	H···*A*	*D*···*A*	*D*—H···*A*
N1—H1C···O1	0.89	2.30	3.000 (4)	135
N1—H1C···O2	0.89	2.15	2.912 (4)	144
N1—H1D···O3	0.89	2.43	2.980 (5)	121
N1—H1D···O4	0.89	2.03	2.886 (4)	161
N1—H1E···O5	0.89	2.43	3.010 (5)	123
N1—H1E···O6	0.89	2.06	2.870 (4)	151
C1—H1B···O11^i^	0.97	2.54	3.398 (9)	148
C13—H13A···O8	0.97	2.52	3.288 (7)	135
C13—H13B···O1^ii^	0.97	2.54	3.504 (6)	171
C15—H15B···O10^iii^	0.96	2.47	3.370 (9)	156

Symmetry codes: (i) −*x*+1, *y*+1/2, −*z*+1/2; (ii) −*x*+1, *y*−1/2, −*z*+1/2; (iii) −*x*, *y*+1/2, −*z*+1/2.
